# Pits, a protein interacting with Ttk69 and Sin3A, has links to histone deacetylation

**DOI:** 10.1038/srep33388

**Published:** 2016-09-13

**Authors:** Gwo-Jen Liaw

**Affiliations:** 1 Department of Life Sciences and Institute of Genome Sciences, National Yang-Ming University, Taipei, 112-22, Taiwan, Republic of China

## Abstract

Histone deacetylation plays an important role in transcriptional repression. Previous results showed that the genetic interaction between *ttk* and *rpd3*, which encodes a class I histone deacetylase, is required for *tll* repression. This study investigated the molecular mechanism by which Ttk69 recruits Rpd3. Using yeast two-hybrid screening and datamining, one novel protein was found that weakly interacts with Ttk69 and Sin3A, designated as Protein interacting with Ttk69 and Sin3A (Pits). Pits protein expressed in the early stages of embryos and bound to the region of the *tor* response element *in vivo*. Expanded *tll* expression patterns were observed in embryos lacking maternal *pits* activity and the expansion was not widened by reducing either maternal *ttk* or *sin3A* activity. However, in embryos with simultaneously reduced maternal *pits* and *sin3A* activities or maternal *pits, sin3A* and *ttk* activities, the proportions of the embryos with expanded *tll* expression were significantly increased. These results indicate that all three gene activities are involved in *tll* repression. Level of histone H3 acetylation in the *tll* proximal region was found to be elevated in embryo with reduced these three gene activities. In conclusion, Ttk69 causes the histone deacetylation-mediated repression of *tll* via the interaction of Pits and Sin3A.

Eukaryotic cells have evolved extremely sophisticated means of regulating and fine-tuning expression of genes in response to various stimuli. Transcriptional activators and repressors play key roles in these activities to control gene expression. In addition, enzymes catalyse acetylation and deacetylation of the core histones and work closely with these transcription factors, as well as with various co-factors, to dynamically change chromatin status from open to closed and vice versa. Chromatin status correlates well with the activation and repression of transcription. In open chromatin, acetylation of the amino-termini of the histones neutralizes the positive charge of these amino acid residues, which results in loose contact between DNA and the nucleosome. When this occurs, transcriptional activators can easily access the appropriate binding sites, and genes are actively transcribed. In contrast, in closed chromatin, the amino-termini of the histones are hypoacetylated and genes are silenced^1^. Consistent with this paradigm, HATs are recruited by transcriptional activators to increase the acetylation level of local chromatin, whereas HDACs are recruited by transcriptional repressors to diminish local acetylation^2^,^3^. Both HATs and HDACs are associated with scaffold proteins and form large multiprotein complexes^4^,^5^.

Scaffold proteins associate with various proteins to coordinate their functions in various cellular processes^6^. Sin3A is one of these scaffold proteins and contains four highly conserved paired amphipathic helix domains, PAH1 to PAH4. The functions of these PAH domains are conserved from yeast to human. For example, a region in PAH3 is known to interact with HDACs. Furthermore, PAH1 and PAH2 bind a variety of transcriptional repressors, as well as co-repressors, and also assist in transcriptional repression in eukaryotes^7^. Components forming the core of the HDAC/Sin3A complexes include HDAC1 (Rpd3 in yeast and fly), HDAC2, RbAp46, RbAp48^8^, RBP1^9^, and/or p33ING1b^10^. Sin3A also binds to a number of docking proteins, e.g. SAP30, SAP18, and SAP25^11^. Because the HDAC/Sin3A complex lacks the ability to bind DNA, it must associate with DNA sequence-specific repressors to function. These repressors include Mad1, E2F-4, MeCP2, ELK1, and KLF^12^,^13^. The recruitment of the HDAC/Sin3A complex by these repressors triggers transcriptional repression via deacetylation and the remodeling of local chromatin into the closed status in the vicinity of the repressor cognate sites^14^. We have shown that Ttk69, but not Ttk88 that is an alternatively spliced product of the *ttk* gene, participates in *tll* repression^15^. Additionally, the genetic interaction between *ttk* and *rpd3* is required for the repression^16^. However, the mechanism by which Rpd3 is recruited is unclear.

Ttk69 is a co-repressor that forms a complex with Hsf and GAGA factor (GAF), and this complex binds to the *tor* response element (*tor*-RE) in the *tll* proximal region^16^,^17^. Ttk69 contains a BTB domain and a zinc-finger motif at its N-terminus and C-terminus, respectively^18^. Ttk69 binds to TCCT elements to regulate the spatial and temporal expression of the *eve, h, odd, run* and *tll* genes during *Drosophila* embryogenesis^15^,^19^,^20^,^21^. To investigate how Ttk69 recruits Rpd3, yeast two-hybrid screening and database mining were used to find a novel protein interacting with both Ttk69 and Sin3A. The protein was designated as Protein interacting with Ttk69 and Sin3A (Pits). Mutants deficient in *pits* expression were generated to reveal its role in *tll* repression. Dosage-dependent genetic interaction experiments were utilised to determine that the genetic interactions of *pits* with *ttk* and *sin3A* are important for *tll* repression. Furthermore, chromatin immunoprecipitation (ChIP) was used to show that the level of histone acetylation is increased in the *tll* proximal region in embryos with reduced *pits, sin3A* and *ttk* activities. These results support the possibility that Pits is a novel mediator linking Ttk69 to histone deacetylation via protein-protein interactions between Pits, Sin3A and Ttk69.

## Results

### CG11138 interacts with both Ttk69 and Sin3A

To explore how Ttk69 recruits Rpd3, the yeast-two hybrid system was utilised to identify Ttk69 interacting proteins. Starting with 34 newly isolated interacting proteins, attempts to find a linkage of Ttk69 with Rpd3 failed. Then, marginally interacting proteins that were histidine positive but mostly β-galactosidase negative were picked to mine protein-protein interaction databases. The data in BioGRID showed that CG11138 interacts with not only Ttk69, but also Sin3A (http://thebiogrid.org/).

**Figure 1:**
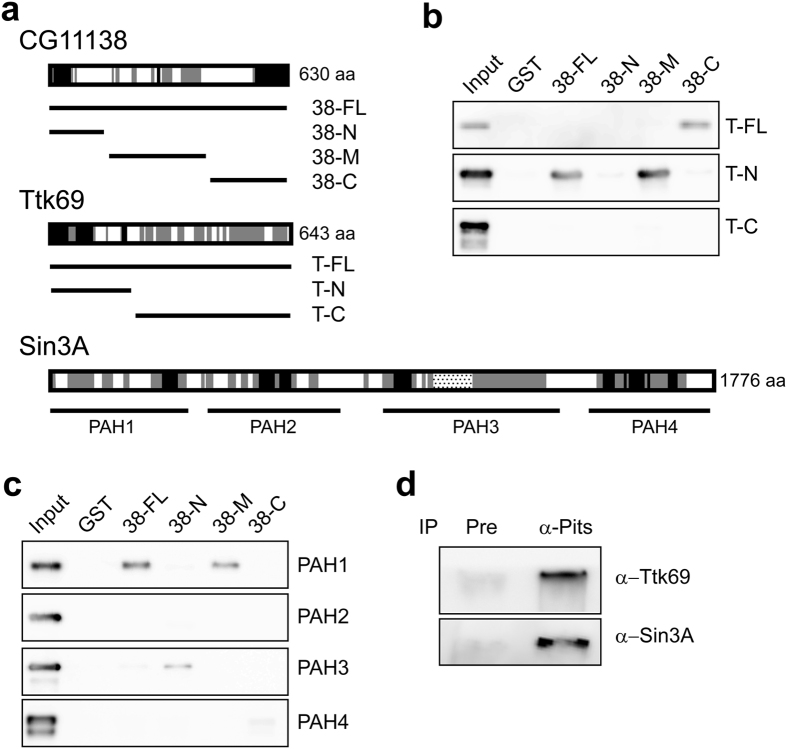
CG11138 interacts with both Ttk69 and Sin3A. (**a**) The shaded and black boxes indicate amino acid residues that are identical among *Drosophila* species and insects, respectively. The dotted box in Sin3A represents the domain that interacts with HDACs. For CG11138 and Ttk69, full-length (FL) and three protein fragments, N, M or C, as indicated by bars below the diagram. The four highly conserved domains in Sin3A, PAH1 to PAH4, as previously described^22^, were expressed in bacteria. (**b**,**c**) Crude extracts containing the GST fusion proteins shown at the top of the panels, were mixed with crude extracts containing one of the S-tag fusion proteins, except for the full-length of Ttk69, which was His-tagged. The proteins were pulled down by glutathione agarose and detected by western blotting using either anti-S tag or anti-His tag antibody. Protein samples, consisting of 10% of the sample used for the pull-down assay, were used as the controls and are designated as Input. (**d**) Ttk69 and Sin3A in a cross-linked protein complex immunoprecipitated by an anti-Pits antibody. The co-IPed protein complexes were separated in SDS agarose-polyacrylamide gels and proteins in the complexes were detected by western blotting with an anti-Ttk69 antibody. The membrane was then stripped to allow detection of Sin3A.

**Figure 2:**
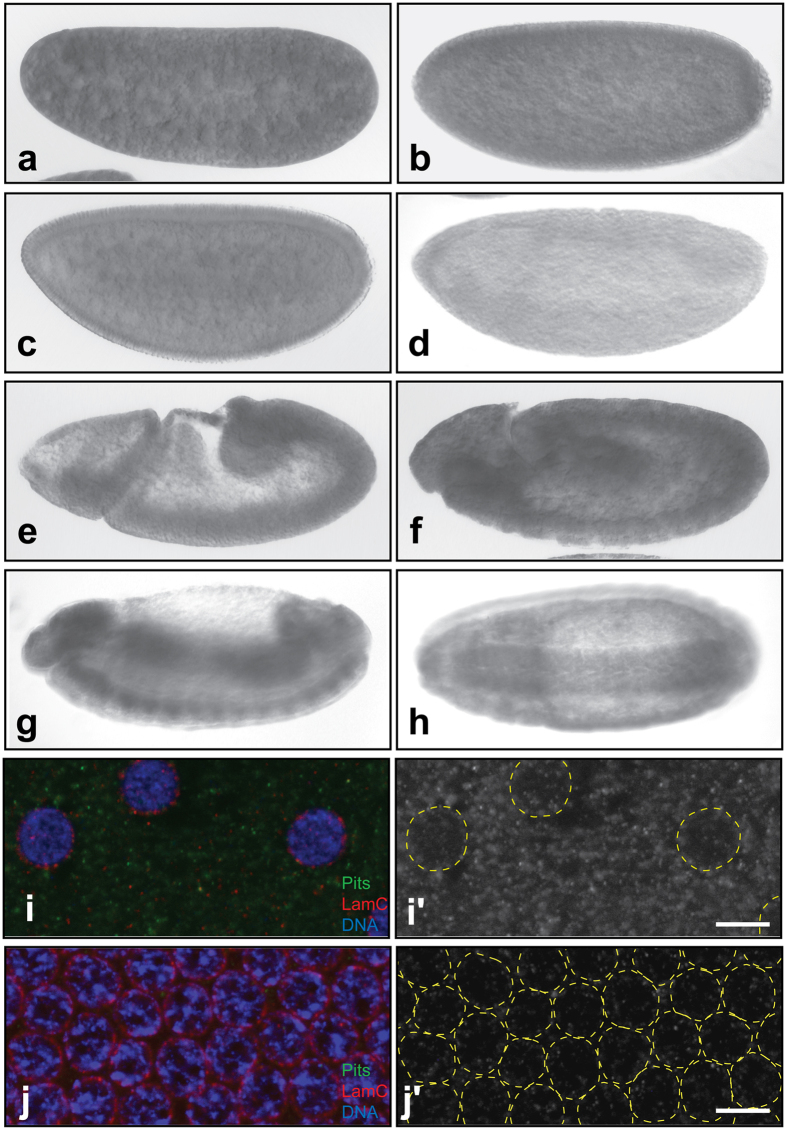
Pits distribution patterns in *Drosophila* embryo. A preabsorbed anti-Pits antibody was used to detect Pits distribution in *Drosophila* embryos by immunostaining. Embryos at stage 3 (**a**), stage 4 (**b**,**i**,**i**’), stage 5 (**c**,**j**,**j**’), stage 6 (**d**), stage 9 (**e**), stage 10 (**f**) and stage 13 (**g**,**h**) are shown. The anterior is displayed to the left. Except for panel h, which shows a ventral view, all panels show a sagittal view of the embryo. (**i**,**j**) To reveal whether Pits is in the nucleus, wild-type embryo was stained with the pre-absorbed anti-Pits (green) and anti-LamC (red) antibodies, and observed using confocal microscopy. Chromosome was labeled with Hoechst 33342 (blue). Punctate staining of Pits was detected in early stage 4 (**i**,**i**’) and 5 embryos (**j**,**j**’). Dashed yellow circles in panels i’ and j’ represent regions of embryonic nuclei to clearly show Pits nuclear localization. Scale bars are 5 μm.

To verify these protein-protein interactions, pull-down experiments were performed. Full-length of Ttk69 (T-FL) might undergo a conformational change because the N-terminal His-tag on T-FL was detected using an anti-His tag antibody (Fig. 1b), but failed to bind Ni-affinity resin (data not shown). Importance of this putative conformational change is discussed later. To explore this possibility, it was essential to use various fragments of the protein. The amino-acid conservation shown in the UCSC genome browser (http://genome.ucsc.edu/) served as a basis for selecting such fragments of Ttk69 (Fig. 1a). Sin3A is a large protein (Fig. 1a), and Zhao *et al*. have reported that it is difficult to synthesize the full-length Sin3A in bacteria^22^. Therefore, using the same rationale, the four PAHs were synthesized. The results from pull-down experiments showed that T-FL weakly interacts with the C-terminus of CG11138 (38-C), but not with full-length CG11138 (38-FL). The N-terminal portion of Ttk69 (T-N) was found to interact with both 38-FL and the middle portion of CG11138 (38-M) (Fig. 1b), supporting the above hypothesis. Furthermore, both 38-FL and 38-M interacted with PAH1. Other portions of CG11138, 38-N and 38-C were found to associate with PAH3 and PAH4 very weakly (Fig. 1c). These results indicated that multiple regions in CG11138 bind weakly to various PAHs and Ttk69. To determine that these three proteins exist in the same protein complex, a co-immunoprecipitation experiment with an anti-Pits antibody was performed. Considering the weak interactions between these three proteins and the relatively low quantity of Pits in the nucleus (Fig. 2i,j), proteins in nuclear extracts were cross-linked by bismaleimidohexane (BMH) before the immunoprecipitation. As shown in Fig. 1d, both Ttk69 and Sin3A were in the same protein complex immunoprecipitated by anti-Pits antibody. These results are consistent with information presented in BioGRID whereby CG11138 directly interacts with both Ttk69 and Sin3A to form a protein complex. Thus, CG11138 was renamed as Pits.

### Pits protein expresses in the early stages of the embryo and later presents at high levels in midgut and central nervous system (CNS)

The data in FlyExpress show uniform *pits* mRNA in embryos from stages1 to 5. At stage 9, *pits* mRNA is enriched in the midgut primordium (http://www.flyexpress.net/). To reveal whether Pits protein follows the same patterns, an anti-Pits antibody was raised and affinity purified. Immunostaining results revealed a high level of Pits in embryos at stages 3 and earlier (Fig. 2a), supporting the possibility that *pits* mRNA is deposited into the egg during oogenesis. This type of mRNAs encodes regulators to control embryogenesis in the early embryonic stages, known as maternal gene activity. Pits protein gradually declined to an undetectable level from stages 3 to 6 (Fig. 2b–d) and then reappeared at stage 8 (Fig. 2e). Later, from stages 10 to 13 (Fig. 2f,g), Pits protein was detected at high levels in midgut and CNS. The midgut and CNS localizations are consistent with the mRNA patterns shown in FlyExpress and the YFP-tagged protein patterns^23^.

**Figure 3:**
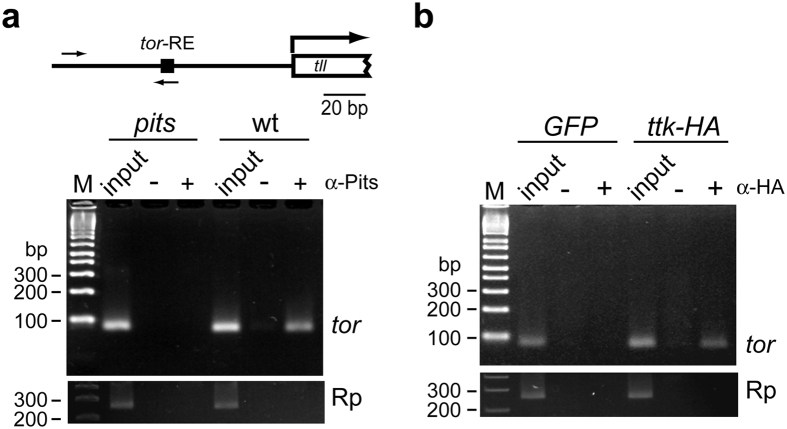
Pits and Ttk69 co-localize to the vicinity of the *tor*-RE *in vivo*. At the top of panel a, a diagram shows the relative positions of the transcription unit of *tll*, the *tor*-RE and a set of primers that is represented by arrows. Embryos were collected from parents of Oregon-R/*w*^*1118*^ (wt) or *pits*^*94*^ (*pits*) (**a**) or *ttk*^*1e11*^ *da-GAL4*/+ *da*-*GAL4* females crossed with either *UAS-GFP (GFP*) or *UAS-ttk69-HA (ttk-HA*) males (**b**) every 2 hours. Embryos aged for 30 min (**a**) or 60 min (**b**) were used in ChIP experiments with anti-Pits (“+”; 6 μg) or anti-HA tag antibody (“+”; 3 μg). Chromatin samples, consisting of 5% of the sample used for ChIP, were used as “input” controls. Mock control, “−”, used the same experimental procedure without antibody. The primer set, *tor*, was used to reveal whether Pits or Ttk69-HA exists in the vicinity of the *tor*-RE. Rp is a set of primers to detect the *RPII140* gene that serves as a PCR negative control. The PCR products were separated in a 4% agarose gel, followed by ethidium bromide fluorography.

Because Pits is a candidate co-repressor for *tll*, it should exist in the nucleus. To reveal whether Pits localizes to the nucleus, immunostaining with confocal microscopy was performed. The results showed 1) punctate patterns uniformly distributed in embryonic cytoplasm at stages 5 and earlier, and 2) lower number of Pits in nuclei, relative to the number in the cytoplasm (Fig. 2i,j). This lower level of Pits in the nucleus opposed its possible role in *tll* repression. However, two lines of evidence still supported its involvement in this process. The first was the results of ChIP experiments, which indicated that both Pits and Ttk69 associate with the *tor*-RE (Fig. 3). The second was that Pits protein contains two domains that are highly homologous to the zinc finger in IRF2BP and the RING finger (Supplementary Fig. S1). Proteins containing either domain repress expression of genes^24^,^25^. Taken together with the interactions between Pits, Ttk69 and Sin3A *in vitro*, Pits might be involved in *tll* repression.

### The maternal *pits* activity is important for *tll* repression

**Figure 4:**
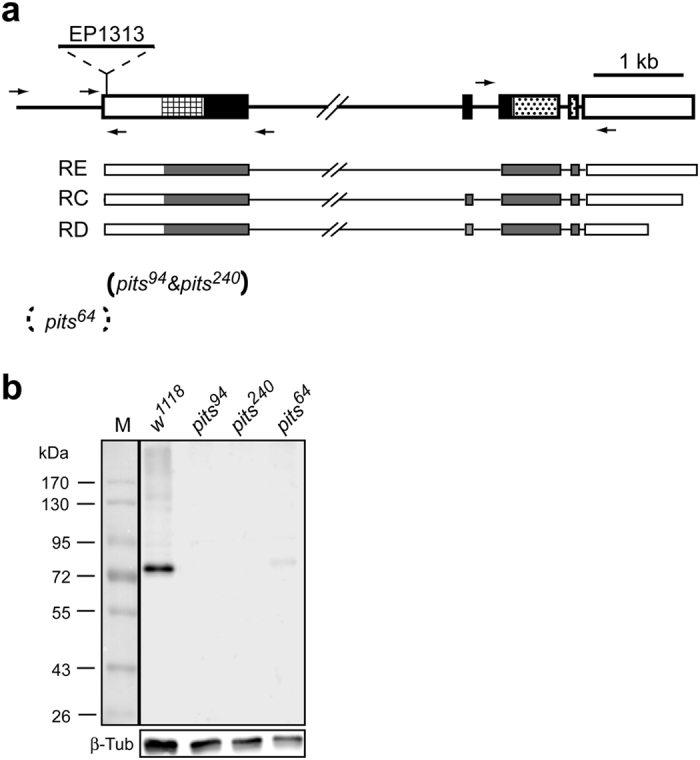
Structure and alleles of the *pits* gene. (**a**) Exons are represented by boxes. Three subregions of Pits, N, M and C as shown in Fig. 1a, are shown by grid, solid and dotted rectangles. The *pits* gene encodes three putative transcripts, represented by RC, RD and RE. Transcripts C and E are the result of alternative splicing inside exon 2. Furthermore, the 3’ untranslated regions in these three transcripts are different. Using the imprecise P-element excision method and a P-element line, EP1313 inserted 38 bp downstream of the putative transcription initiation site for the transcripts, three *pits* deletions, 64, 94 and 240, were obtained. The arrows indicate the positions and direction of the primers used for screening the *pits* deletions. The range of each deletion, from position +39 to +1621, is indicated by brackets. The dashed bracket indicates that the 5′ end of the deletion has not been determined. (**b**) Pits levels in *w*^*1118*^, *pits*^*94*^, *pits*^*240*^ and *pits*^*64*^ embryos from 0 to 4 hours were assessed by western blotting with an anti-Pits antibody. The membrane was then stripped to allow detection of β-Tubulin (β-Tub), which served as the loading control. M represents the protein size marker.

To test the above hypothesis, *pits* deletions were generated using the imprecise P-element excision method with EP1313, which is inserted 38 bp downstream of the putative transcription initiation site (Fig. 4a). Two *pits* alleles, 94 and 240, were obtained that had identical molecular lesions where exon 1 was almost deleted (Fig. 4a). The transcription initiation site remained intact in both deletions. Surprisingly, both alleles are homozygote viable with no observable phenotype. Another *pits* allele, 64, was found to be semi-lethal. No PCR product was produced when the upstream primers were used (Fig. 4a), suggesting that the lethality results from truncation of one or more genes upstream of *pits*.

To explore Pits levels in *pits*^*94*^, *pits*^*240*^ and *pits*^*64*^ embryos in the early stages, western blotting was performed. The results revealed a ~75 kDa protein in *w*^*1118*^ embryo with a minor band, just below the major band, also visible (Fig. 4b). These two bands were presumably the products translated from transcripts C and E (see legend of Fig. 4), despite the fact that both proteins were larger than the calculated molecular weights of 60 and 62 kDa. Although no Pits protein was detected in *pits*^*94*^ or *pits*^*240*^ embryos (Fig. 4b), perhaps, both *pits*^*94*^ and *pits*^*240*^ are not null alleles. The lethality of embryos (38.0%) obtained from *pits*^*94*^/*Df*(*X*)*BSC624* females crossed with *pits*^*94*^ males was much higher than those from *pits*^*94*^ or *pits*^*240*^ parents (approximately 3.5%). The difference might result from that an open reading frame in the truncated *pits* mRNA encodes a truncated polypeptide containing 29% of 38-M and intact 38-C, which may have residual Pits activity, and that quantity of the truncated Pits is too low to be detected by western blotting. Alternatively, a gene within the deleted region of *Df*(*X*)*BSC624* may contribute the phenotype, which is similar to the finding of the *sad*^1^ deletion^26^. Residual Pits in *pits*^*64*^ embryos (Fig. 4b) further supported the possibility that the lethality of *pits*^*64*^ was due to defects in one or more other gene(s).

**Figure 5:**
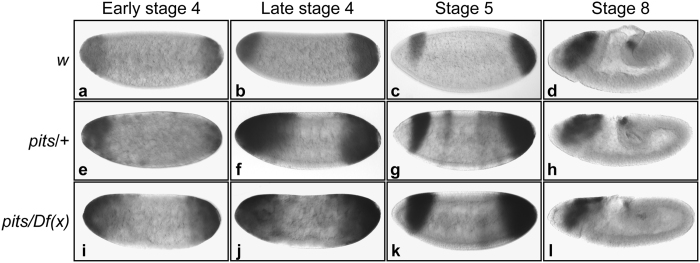
Maternal *pits* activity is important for *tll* repression. The *tll* expression patterns in embryos from *w*^*1118*^ (**a**–**d**), a cross of *pits*^*94*^ with *w*^*1118*^ (*pits*/+; **e**–**h**) and a cross of *pits*^*94*^/*Df(X)BSC624* with *pit*^*94*^ (*pits*/*Df(X)*; **i–l**) were determined by *in situ* hybridization using digoxigenin-labeled antisense *tll* RNA as the probe. The embryonic stages are indicated at the top of the panels. Embryos are arranged in a sagittal view, with the anterior towards the left.

**Figure 6:**
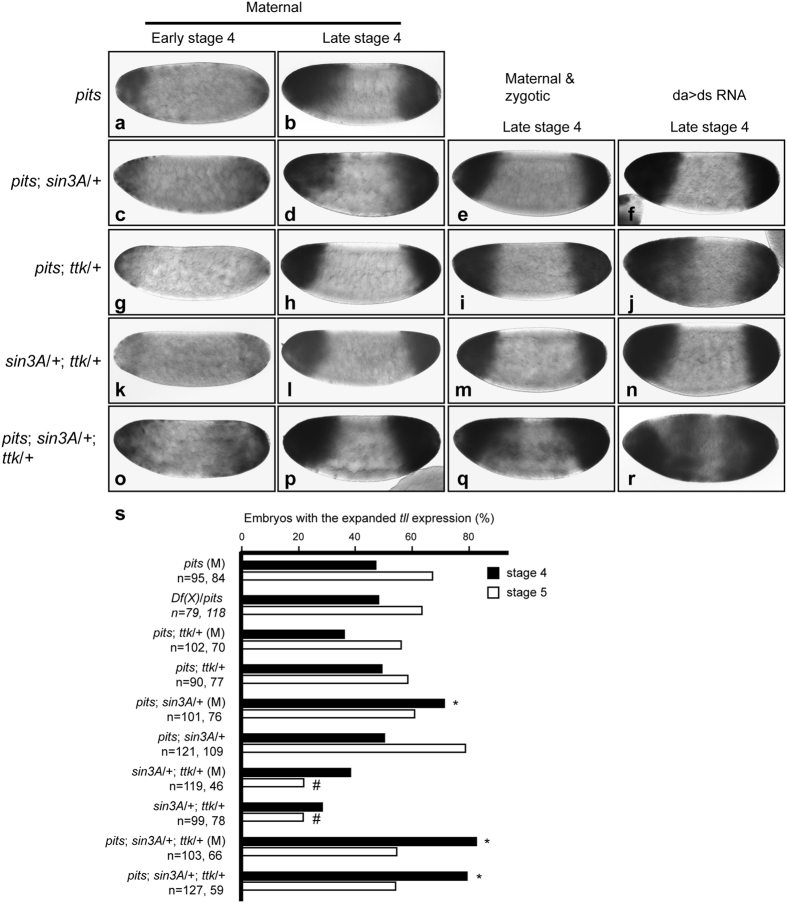
The genetic interactions of *pits* with *sin3A* and *ttk* are important for *tll* repression. To test the maternal effect of the gene activities, embryos from females *pits*^*94*^ (**a**,**b**), *pits*^*94*^; *sin3A*^*ex4*^/+ (**c**,**d**), *pits*^*94*^; *ttk*^*1e11*^/+ (**g**,**h**), *sin3A*^*ex4*^/+; *ttk*^*1e11*^/+ (**k**,**l**) and *pits*^*94*^; *sin3A*^*ex4*^/+; *ttk*^*1e11*^ (**o**,**p**) crossed with *w*^*1118*^ males were collected to determine *tll* expression patterns. To test the maternal and zygotic effect of the genes’ activities, females and males that had the same genotypes, as shown at the left, were mated (**e**,**i**,**m**,**q**). Due to undetectable GFP protein expressed by the *ubi*-*GFP* transgene in the balancer chromosome in early embryonic stages, genotypes of embryos homozygous for *sin3A* or *ttk* could not be determined. Therefore, RNAi was used. Females carrying the *da-GAL4* transgene were mated with RNAi lines to knock down at least two gene activities simultaneously (**f**,**j**,**n**,**r**). The *tll* expression patterns in the embryos were revealed by *in situ* hybridization with digoxigenin-labeled antisense *tll* RNA. Embryos are arranged in a sagittal view, with the anterior towards the left. (**s**) A bar graph presents percentages of embryos with expanded *tll* expression patterns. “M” in brackets represents embryos only with reduced maternal gene activities. Percentage indicates proportion of embryos with expanded *tll* expression patterns over the total number of stage-4 (solid bars) or stage-5 (open bars) embryos. The left and right numbers beneath each genotype are the total numbers of stage-4 and stage-5 embryos. *Df(X)* represents *Df(X)BSC624*. Fisher’s exact test was used to determine statistical significance of proportion of embryos with the expanded *tll* expression from *pits* (M) mothers or *Df(X)*/*pits* crossed with *pits* males against those with further reduction of one or two more gene activities (*: increase, #: decrease, *p* < 0.001).

To explore whether maternal *pits* activity is important for *tll* repression, embryos were collected from *pits*^*94*^ females crossed with *w*^*1118*^ males. Then, *tll* expression patterns were investigated by *in situ* hybridization with digoxigenin-labeled antisense *tll* RNA probe. In 47% of embryos with reduced maternal *pits* activity, *tll* expression patterns were slightly expanded towards the central region of embryos (Figs 5f and 6s). The expanded patterns had gradually returned to normal by stage 8 (Fig. 4h) when compared with wild-type embryos (Fig. 5d). The degree of expansion of the *tll* expression pattern was similar in embryos from *pits*^*94*^ parents (data not shown) and embryos from *pits*^*94*^/*Df(X)BSC624* females crossed with *pits*^*94*^ males (Figs 5i–l and 6s). These results supported the idea that maternal *pits* activity is important for *tll* repression, and suggested that zygotic *pits* activity contributes less in *tll* repression, which is consistent with the lack of Pits protein in embryos from late-stage 5 to stage 7 (Fig. 2).

### Maternal *pits, sin3A* and *ttk* activities work together to repress *tll* expression

The results described above indicated that maternal *pits* activity is involved in *tll* repression. To test whether maternal *pits* interacts genetically with maternal *sin3A* and/or *ttk* to repress *tll* expression, the dose dependent genetic interaction experiment was performed. Embryos were collected from females that had activities of two or three of the relevant genes reduced and then crossed with *w*^*1118*^ males. These embryos were used to determine *tll* expression patterns. The results showed that the percentage of embryos with the expanded *tll* expression significantly increased from 47% in *pits*^*94*^ to 71% in *pits*^*94*^; *sin3A*^*ex4*^/+ (Fig. 6s). Hereafter, percentage is used to indicate embryos with the expanded *tll* expression. Although there were reduced percentages of *pits*^*94*^; *ttk^1e11^*/+ and *sin3A*^*ex4*^/+; *ttk^1e11^*/+ embryos (36% and 39%, respectively; Fig. 6h,l,s), unexpectedly, the difference relative to *pits*^94^ embryos were not significant (Fig. 6b,s). These results countered the hypothesis that interactions between these genes are involved in *tll* repression. Nevertheless, a simultaneous reduction of the maternal *pits, sin3A* and *ttk* activities significantly increased the percentage of the embryos (82%, Fig. 6p,s), compared to that of *pits*^94^ embryos. These results supported the hypothesis that the maternal activities of *pits, sin3A* and *ttk* work together to repress *tll* expression.

Sin3A is a widely distributed factor^27^. The zygotic activities of the above genes might function in *tll* repression in spite of the facts that both Pits and Ttk69 are not detected in late stage-5 embryo^15^. To test this possibility here, *tll* expression patterns in embryos from parents of *pits*^*94*^; *sin3A*^*ex4*^/+, *pits*^*94*^; *ttk^1e11^*/+, *sin3A*^*ex4*^/+; *ttk^1e11^*/+ or *pits*^*94*^; *sin3A*^*ex4*^/+; *ttk^1e11^*/+ were examined. Because the maternal-zygotic transition occurs at late stage 4, effects on *tll* expression patterns at either late stage 4 or early stage 5 should result from both maternal and zygotic gene activities. The results showed no significant changes in the degree of expansions in these embryos (Fig. 6e,i,m), compared to those with reduced maternal gene activities (Fig. 6d,h,l). Unexpectedly, the reduction of both maternal and zygotic *ttk* and *sin3A* gene activities led to an exceptionally low percentage of embryos (29%, Fig. 6m,s). This may be due to dual functions of *Ttk69* and *Sin3A* (see Discussion). Again, the expanded *tll* expression in embryos from *pits sin3A ttk* parents was observed in the middle region of the embryos, compared to those with reduced maternal activities only (Fig. 6p,q). The percentages of embryos significantly increased as 79% (Fig. 6s). In all cases, the expanded *tll* expression patterns returned to normal by stage 10 (Supplementary Fig. S2). These results indicated that the zygotic activities of these genes contributed less to *tll* repression.

Because it is difficult to identify embryo genotypes, RNAi was used to verify the results from the genetic interaction experiments. The rationale for this experiment was that maternally provided GAL4 from the *da-GAL4* transgene drives the synthesis of double stranded RNA at early stage 4, which consists of the timing of zygotic gene expression and presents the beginning of the stage lasting 50 minutes. Hypothetically, all targeted mRNAs are knocked down by late stage 4, but not early stage 4. Therefore, females carrying the *da*-*GAL4* transgene were crossed with males carrying at least two *UAS*-RNAi transgenes. *tll* expression patterns were only observed at late stage 4 and early stage 5. In all cases, degree of the expanded *tll* expression patterns were increased (Fig. 6f,j,n,r vs. Fig. 6e,i,m,q). Consistently, the percentages of embryos were similar to those with reduced maternal and zygotic gene activities. These data supported the conclusion that interactions between maternal *pits, sin3A* and *ttk* gene activities are important for attenuating *tll* expression.

### An increased level of histone H3 acetylation in the *tll* proximal region is present when there is reduced *pits, sin3A* and *ttk* activity

**Figure 7:**
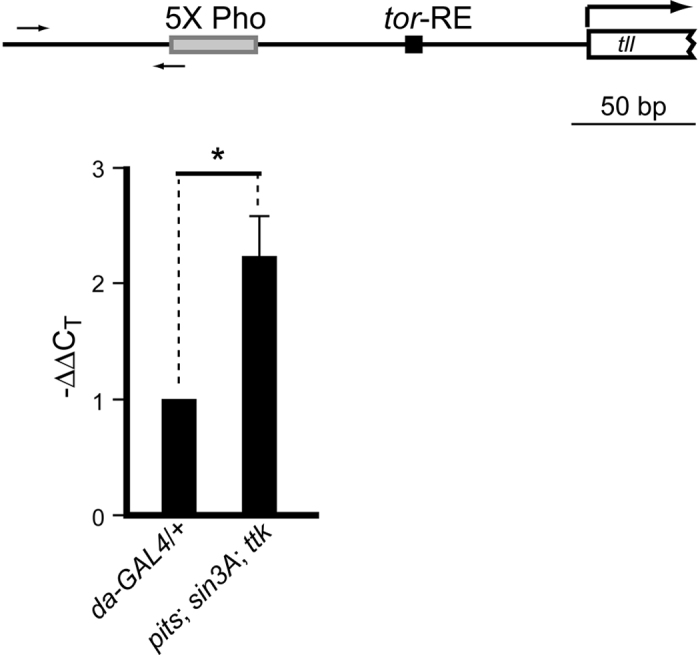
Simultaneous reduction of *pits, sin3A* and *ttk* activity increases the level of histone acetylation in the *tll* proximal region. Embryos were collected from *da-GAL4* females crossed with *w*^*1118*^ males (*da-GAL4*/+) or *pits*^*94*^/+; *sin3A*^*ex4*^/+; *ttk*^*1e11*^ *da-GAL4*/*daGAL4* females crossed with males carrying multiple transgenes to knock down *pits, sin3A* and *ttk69* mRNA (*pits; sin; ttk*). Embryos from these crosses were used to determine the level of histone acetylation in region adjacent to the *tor*-RE by ChIP with an anti-acetyl histone H3 specific antibody (Merck Millipore). A cluster of Pho binding sites, called 5X Pho that is 173 bp upstream the *tor*-RE, is at the 5′ end of a putative PRE^40^. Arrows indicate a set of primers used for real-time PCR. Detection of *act-5C* served as an endogenous control. C_T_ values of *act* were used to normalize *tll*, designated as ΔC_T_. Relative amounts are represented by −ΔΔC_T_ where the ΔC_T_ value for the *pits; sin; ttk* embryos are subtracted from that of the control embryos. Significance difference was determined by Student’s *t*-test (*p < 0.05).

Our previous and current results have indicated that the genetic interactions of *ttk69* with *rpd3* and *sin3A* are important for *tll* repression^16^, which suggests that Rpd3 is recruited by Ttk69 through Pits and Sin3A to deacetylate histones and results in an attenuation of *tll* expression. Thus, histone acetylation in the region adjacent to the *tor*-RE ought to be increased in embryos that have reduced *pits, sin3A* and *ttk* activity. To test this possibility, the level of histone acetylation in the *tll* proximal region was determined by ChIP using an antibody against histone H3 that is acetylated at Lys9 and Lys14. The results showed that the relative amount of acetylated histone H3 in the vicinity of the *tor*-RE is increased by 4.7-fold in embryos with reduced *pits, sin3A* and *ttk69* activity (2^2.23^-fold; Fig. 7). These results supported the hypothesis that histone deacetylation is involved in *tll* repression. In conclusion, the above findings show that the Ttk69 co-repressor recruits the Rpd3/Sin3A complex to exert histone deacetylation-mediated transcriptional repression of *tll*.

## Discussion

**Figure 8:**
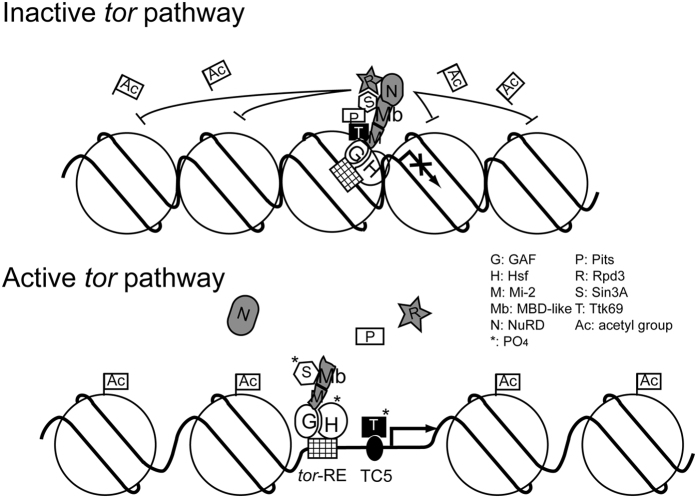
A model of Pits associating with Rpd3 and Mi-2/NuRD complexes with and without stimulation of the active *tor* pathway. Previous results showed that GAF, Hsf and Ttk69 form a protein complex that binds to the *tor*-RE (grid rectangle). *tll* expression is attenuated in the middle of embryo where *tor* is inactive^16^. Here, Pits serves as a mediator to recruit the Rpd3/Sin3A complex. In addition, based on information from the literature, GAF likely recruits the Mi-2/NuRD complex (indicated by shaded diagrams)^30^. Both Rpd3 and NuRD complexes associate with Ttk69 and GAF to inhibit histone acetylation (Ac) in the *tll* locus. At both poles of *Drosophila* embryo, Erk activated by the active *tor* pathway phosphorylates Ttk69, Sin3A and Hsf, indicated by asterisks. The phosphorylated Hsf becomes an activator, whereas the phosphorylated Ttk69 is released from the protein complex, leading to the disruption of the association of the Rpd3 and NuRD complex with the *tor*-RE, and also converts to an activator that binds to TC5. Previous work has shown that Sin3A is phosphorylated by Erk and converts it to an activator^34^. Therefore, phosphorylated Sin3A may associate with Mi-2/MBD-like proteins to activate *tll* expression.

Pits plays an important role in Ttk69-mediated recruitment of the Rpd3/Sin3A complex to attenuate *tll* expression. When initiating *tll* repression at early stage 4, Ttk69 acts as a co-repressor that increases the ability of the GAF/Hsf heterodimer to bind to the *tor*-RE^16^. The Rpd3/Sin3A complex is then brought to the *tor*-RE via the interaction of Pits with both Ttk69 and Sin3A. As a result, the level of acetylated histone H3 in the vicinity of the *tor*-RE decreases in the middle region of embryo (top panel in Fig. 8). This hypoacetylation is consistent with the data in modENCODE (http://gbrowse.modencode.org/fgb2/gbrowse/fly/), which shows no acetylation or low level on Lys9 and Lys27 of histone H3 from position −550 to +160 in the *tll* gene. In addition, Rpd3, HDAC3 and HDAC6 are detected in this region *in vivo* (modENCODE). This raises the question as to which enzyme executes the deacetylation. The data in the modENCODE development RNA-Seq database show that the level of *rpd3* mRNA is significantly higher than those of *HDAC3* and *DHAC6* mRNAs in 0–4 hr embryos^28^. Therefore, in the early stages of *Drosophila* embryo, Rpd3 is the major enzyme to execute histone deacetylation, which results in the chromatin status of the *tll* gene switching to the closed configuration. In turn, this conformational change results in attenuation of *tll* expression.

In addition to Pits, Ttk69 together with GAF interacts with other co-repressors that can recruit Rpd3. First, GAF interacts with SAP18, a docking protein in the Rpd3/Sin3A complex^29^. GAF, SAP18, Sin3A and Rpd3 co-localize to a Polycomb repression element, *Fab-7*. Reduction of these gene activities results in histone hypoacetylation on *Fab-7* that reduces its function in gene silencing^30^. Secondly, GAF and Ttk69 associate with the Mi-2/NuRD chromatin remodeler complex (NuRD) that also contains Rpd3^31^ (top panel in Fig. 8). Compensation by these redundant factors may therefore result in less expanded *tll* expression patterns indicated by lower levels of involvement of the zygotic activities in *tll* repression.

An opposite effect was observed in which the percentage of *sin3A*/+*; ttk*/+ embryos is significantly lower than others. Regardlessly, this may be similar to what occurs with *stg* expression that is up- or down-regulated by *ttk69* overexpression or by *sin3A* knock-down. These may result from indirect inhibition of a *stg* activator^32^. Here, it is difficult to fully elucidate the opposite effect coming from indirect inhibition to expression of a *tll* activator. When considering NuRD involved in *tll* repression, it provides a better scenario to explain the opposite effect. Previous work demonstrated that SUMOylated LIN-1 acts as a repressor through NuRD, whereas LIN-1 phosphorylated by Erk becomes an activator and that NuRD is able to activate expression of certain genes^33^,^34^. In addition, Erk converts Sin3A to an activator by phosphorylation^35^. Although Ttk69 is a transcription repressor, similarly, it also cooperates with REPO to activate expression of the M84 marker^36^. Thus, at both poles of embryo, Erk activated by the active *tor* pathway phosphorylates Ttk69 and Sin3A. Phosphorylated Ttk69 binds to the cognate binding sites (TC5) flanking the *tor*-RE^15^,^16^ to activate *tll* expression and phosphorylated Sin3A retained by Mi-2/MBD-like activates *tll* expression (bottom panel in Fig. 8). When both Ttk69 and Sin3A are reduced, the repressive activity of Rpd3 and NuRD complexes may be slightly affected because Pits can hold on both complexes associated with the *tor*-RE. A low level of *tll* activation occurs in *sin3A*/+*; ttk*/+ embryos, leading to the lowest percentage. When Pits is also absent, the repressive activity of NuRD may greatly be affected, causing a high level of *tll* expression. Therefore, the embryo shows the highest percentages (Fig. 6q,s; bottom panel in Fig. 8). The involvement of Pits in the Rpd3 and NuRD repressive mechanisms provides insights into how transcription co-repressors fine-tune the expression levels of genes responding to stimuli, such as cellular signals^34^.

Another inconsistent result was observed in this study. The BTB domain (T-N) weakly interacts with 38-FL and 38-M, whereas T-FL weakly interacts with only 38-C. There is a two-part explanation for these results. First, Pits can contact the PAH1 domain in Sin3A to open its C-terminus; then, the free C-terminus of Pits can bind to Ttk69 and cause the release of the N-terminus of Ttk69 from the wrapping. The BTB domain of Ttk69 is an important domain for protein-protein interactions. Aside from interacting with dCtBP^3^ and SMRT^37^, it also strongly interacts with the BTB domains of other members of the Ttk BTB subfamily^38^,^39^ and forms a protein complex. These co-repressor complexes may then function in *tll* repression.

In the proposed model, transcriptional co-repressors play important roles to silence gene expression by remodeling chromatin into a closed status that prevents transcription in eukaryotes. The pleiotropic roles of Sin3A in transcriptional repression have been shown. Sin3A forms the Rpd3/Sin3A complex to deacetylate the histones in the vicinity of a promoter^7^. Furthermore, the Rpd3/Sin3A complex can be expanded by adding extra catalytic modules onto the platform. In addition to NuRD described above, transcriptional modulators include enzymes that catalyze methylation; still others catalyze the O-linkage of monosaccharide N-acetylglucoseamine to histones and various other chromatin remodeling enzymes^7^. These chromatin remodelers play pivotal roles in epigenetic regulation^40^. In the *tll* gene, there is a putative Polycomb repression element (PRE) adjacent to the *tor*-RE^41^. In the present study, it was found that histone deacetylation occurs in the region overlapping the 5′ end of the PRE (Fig. 7). Thus, it is likely that chromatin structure in the *tll* gene is remodeled by the Rpd3/Sin3A complex to facilitate the silencing of posterior *tll* expression and that this occurs by Polycomb epigenetic repression (PER) after stage 6. The finding of Pits/Rpd3/Sin3A in initiating *tll* repression provides a clue for further investigation into how PER is established. This newly elucidated mechanism could increase our understanding of various aspects of metazoan biology, such as development, diseases and ageing.

## Methods

### Fly lines and genetics

Lines *ttk^1e11^*/*TM3 Sb*^*1* ^42 and *sin3A*^*ex4*^/*CyO*^27^, generously provided by Drs. Y.-N. Jan and D.-H. Huang, were used in the genetic studies, described below. Line *UAS-ttk69-HA* provided by Dr. L.C. Lai^3^ was used in ChIP to show the *in vivo* binding of Ttk69 to the *tll* locus. Three RNAi lines, *w*^*1118*^; *P*{*GD7212*}*v18154, w*^*1118*^; *P*{*GD4387*}*v10808*/*CyO* and *w*^*1118*^; *P*{*GD4414*}*v10855* that knock down activities of *pits, sin3A* and *ttk69*, were obtained from Vienna *Drosophila* RNAi Center. Because the *w*^*1118*^; *p*{*GD4387*}*v10808*/*CyO* line has a few escapers, *p*{*GD4387*} was mobilized to obtain lines that are homozygous viable^43^.

*P*{*EP*}*CG11138*^*EP1313*^ from Bloomington Stock Center was used to generate *pits* deletions using imprecise excision method^43^ and polymerase chain reaction (PCR) (locations of primers are shown in Fig. 3a). Molecular lesions were revealed by DNA sequencing the PCR amplified DNA fragments. A deletion mutant, *Df(X)BSC624 w*^*1118*^/*Binsinscy* from Bloomington Stock Center, was used to test whether the newly generated *pits* alleles were null alleles.

### Yeast two-hybrid screening

The coding region of *ttk69* was amplified using PCR with a set of primers and pNB408^44^ as template DNA and inserted into of pAS2-1 (Clontech. Inc. Palo Alto, CA). The resulting plasmid DNA was transformed into a yeast strain Y187. A cDNA library, made from 0–12 hour of *Drosophila* embryo, was generously provided by Dr. C. Chein and transformed into a yeast strain, CG1945. Transformants were pooled to the final concentration of 50 OD_600_. Nine 1-ml aliquots were mated with the *ttk69* transformants^45^. The cDNA inserts were amplified from the histidine positives by PCR and then digested with HaeIII for grouping. One from each group was randomly picked to confirm by analyzing β-galactosidase activity to eliminate false positives, compared to that of a negative control, pAS-Laminin. DNA sequences at the 5’end of the cDNA inserts were then determined and used to identify genes by BLAST.

### Plasmid construction and expression of proteins in *Escherichia coli*

Amino acids conserved among *Drosophila* species and other insects served as bases for subcloning various fragments of Pits, Sin3A and Ttk69 into modified pGEX2T (GE Bioscience) and pET29a (Novagen) (Fig. 1a). Primers are listed in Supplementary Table S1 and used to amplify full-length and various portions of cDNAs in RE41430, LD13852 or pNB408. The PCR amplified DNA fragments were then cloned into the bacterial expression vectors. The GST- or S-tag-fusion proteins were expressed in either *E. coli* DH5α pG-tf2 or *E. coli* BL21 (DE3) pG-tf2 using procedures described by Ausubel *et al*.^46^. The expression of the proteins was monitored by the predicted size in SDS polyacrylamide gel^46^.

The GST-fused 38C was purified using glutathione agarose chromatography (GE Bioscience) and used to raise anti-Pits antibody. Production and affinity purification of anti-Pits antibody were performed by LTK BioLaboratories, Taiwan.

### GST pull-down assays and western blotting

The detection of interactions of Pits with Sin3A or Ttk69 was carried out as previously described^47^.

Protein extracts from *Drosophila* embryos from various parents were prepared and used to determine Pits levels in these embryos. Western blotting was used to detect Pits and the bacterially expressed proteins during pull-down experiments^48^.

### Co-immunoprecipitation

The co-IP procedure was carried out as previously described^49^ with modifications. Nuclei were isolated from 0.5-3-hr embryos^17^, resuspended in a lysis buffer containing 1.5 mM NaH_2_PO_4_, 8 mM Na_2_HPO_4_, 145 mM NaCl, 1 mM MgCl_2_, 10% glycerol, and protease inhibitors (EDTA free, Roche Applied Science). The nuclear debris was removed by centrifugation. Proteins in nuclear extracts were cross-linked by 25 μM of BMH (ThermoFisher Scientific). The cross-linked protein complexes were immunoprecipitated by an anti-Pits antibody described by Liu *et al*.^49^. The co-IPed proteins were separated in 0.5% agarose/3.4% SDS polyacrylamide gel^50^ and detected proteins in the co-IPed protein complexes by western blotting with anti-Ttk69 (affinity purified by GeneTex, Inc. Taiwan) and anti-Sin3A antibodies (Santa Cruz Biotechnology, Inc).

### Immunostaining and *in situ* hybridization

Embryos were collected every 12 hours and fixed by 5% paraformaldehyde in 1X PBS and then incubated with anti-Pits antibody (1:2000)^51^. Because several proteins in *w*^*1118*^ embryonic extract were weakly detected by the anti-Pits antibody (Fig. 4b), the antibody was preadsorbed by *pits* embryos to minimize background of the immunostaining. Pits localizations were revealed by incubating with an anti-rabbit IgG conjugated with alkaline phosphatase and colorimetric substances (5-bromo-4-chloro-3-indolyl phosphate and nitroblue tetrazolium, Roche) or anti-rabbit IgG conjugated with Cy3^51^. *in situ* hybridization was carried out as described previously^52^. Patterns of Pits protein and of *tll* mRNA in embryos were viewed using DIC light microscope (Leica Model DMR) or confocal microscope (Olympus Model FV10).

### Chromatin immunoprecipitation

The ChIP procedure was carried out as previously described^53^ with modifications. Staged embryos were collected to isolate paraformaldehyde-fixed and fragmented chromatin^52^. The fixed nuclei were pelleted by centrifugation at 2000× g. Background of protein-A Dynabeads (Invitrogen) was reduced by pre-treatment and blocking as previously described^54^.

A highly acetylated region in the *act-5C* gene presented in modENCODE was selected to serve as an endogenous control. Primers actin-F and actin-R (Supplementary Table S1) were designed to amplify the acetylated region, position from −190 to −314 in *act-5C*. To reveal whether a reduced level of *pits, sin3A* and *ttk* activity was correlated to an increased level of histone acetylation in the *tll* proximal region, primers tll-F and tll-R (Supplementary Table S1) were used to amplify the cis-regulatory regions of the *tll* gene. The relative quantity of ChIPed chromatin from *tll*, compared to *act-5C*, was determined using StepOne software v2.1 (ABI Biosystems) and SYBR qPCR kit (2x master mix, KAPA Biosystems), represented by ΔC_T_ values. The ΔC_T_ values from *pits, sin3A* and *ttk* embryos were subtracted from those of the control embryos to obtain ΔΔC_T_ values.

To reveal whether Pits or Ttk69 binds to the vicinity of the *tor*-RE, primers tor-RE F and tor-RE R (Supplementary Table S1) were used.

## Additional Information

**How to cite this article**: Liaw, G.-J. Pits, a protein interacting with Ttk69 and Sin3A, has links to histone deacetylation. *Sci. Rep.* **6**, 33388; doi: 10.1038/srep33388 (2016).

## Supplementary Material

Supplementary Information

## References

[b1] Beisel C. & Paro R. Silencing chromatin: comparing modes and mechanisms. Nat. Rev. Genet. 12, 123–135, doi: 10.1038/nrg2932 (2011). 21221116

[b2] Roth S. Y., Denu J. M. & Allis C. D. Histone acetyltransferases. Annu. Rev. Biochem. 70, 81–120 70/1/81, doi: 10.1146/annurev.biochem.70.1.81 (2001). 11395403

[b3] Wen Y., Nguyen D., Li Y. & Lai Z. C. The N-terminal BTB/POZ domain and C-terminal sequences are essential for Tramtrack69 to specify cell fate in the developing *Drosophila* eye. Genetics 156, 195–203 (2000). 10978285 10.1093/genetics/156.1.195 PMC1461259

[b4] Narlikar G. J., Fan H. Y. & Kingston R. E. Cooperation between complexes that regulate chromatin structure and transcription. Cell 108, 475–487 (2002). 11909519 10.1016/s0092-8674(02)00654-2

[b5] Ouyang J., Shi Y., Valin A., Xuan Y. & Gill G. Direct binding of CoREST1 to SUMO-2/3 contributes to gene-specific repression by the LSD1/CoREST1/HDAC complex. Mol. Cell 34, 145–154, doi: 10.1016/j.molcel.2009.03.013 (2009). 19394292 PMC2727917

[b6] Fritz R. D. & Radziwill G. CNK1 and other scaffolds for Akt/FoxO signaling. Biochim. Biophys. Acta 1813, 1971–1977, doi: 10.1016/j.bbamcr.2011.02.008 (2011). 21320536

[b7] Silverstein R. A. & Ekwall K. Sin3: a flexible regulator of global gene expression and genome stability. Curr. Genet. 47, 1–17, doi: 10.1007/s00294-004-0541-5 (2005). 15565322

[b8] Zhang Y. . SAP30, a novel protein conserved between human and yeast, is a component of a histone deacetylase complex. Mol. Cell 1, 1021–1031 (1998). 9651585 10.1016/s1097-2765(00)80102-1

[b9] Lai A. . RBP1 recruits the mSIN3-histone deacetylase complex to the pocket of retinoblastoma tumor suppressor family proteins found in limited discrete regions of the nucleus at growth arrest. Mol. Cell. Biol. 21, 2918–2932, doi: 10.1128/MCB.21.8.2918-2932.2001 (2001). 11283269 PMC86920

[b10] Skowyra D. . Differential association of products of alternative transcripts of the candidate tumor suppressor ING1 with the mSin3/HDAC1 transcriptional corepressor complex. J. Biol. Chem. 276, 8734–8739, doi: 10.1074/jbc.M007664200 (2001). 11118440

[b11] Alland L. . Identification of mammalian Sds3 as an integral component of the Sin3/histone deacetylase corepressor complex. Mol. Cell. Biol. 22, 2743–2750 (2002). 11909966 10.1128/MCB.22.8.2743-2750.2002 PMC133736

[b12] Ayer D. E. Histone deacetylases: transcriptional repression with SINers and NuRDs. Trends Cell. Biol. 9, 193–198 (1999). 10322454 10.1016/s0962-8924(99)01536-6

[b13] Cowley S. M. . Functional analysis of the Mad1-mSin3A repressor-corepressor interaction reveals determinants of specificity, affinity, and transcriptional response. Mol. Cell. Biol. 24, 2698–2709 (2004). 15024060 10.1128/MCB.24.7.2698-2709.2004 PMC371107

[b14] Margueron R. & Reinberg D. Chromatin structure and the inheritance of epigenetic information. Nat. Rev. Genet. 11, 285–296, doi:. doi: 10.1038/nrg2752 (2010). 20300089 PMC3760772

[b15] Chen Y. J., Chiang C. S., Weng L. C., Lengyel J. A. & Liaw G. J. Tramtrack69 is required for the early repression of *tailless* expression. Mech. Dev. 116, 75–83 (2002). 12128207 10.1016/s0925-4773(02)00143-0

[b16] Chen Y. C., Lin S. I., Chen Y. K., Chiang C. S. & Liaw G. J. The Torso signaling pathway modulates a dual transcriptional switch to regulate *tailless* expression. Nucleic acids research 37, 1061–1072, doi: 10.1093/nar/gkn1036 (2009). 19129218 PMC2651784

[b17] Liaw G. J. . The *torso* response element binds GAGA and NTF-1/Elf-1, and regulates *tailless* by relief of repression. Genes Dev. 9, 3163–3176 (1995). 8543159 10.1101/gad.9.24.3163

[b18] Albagli O., Dhordain P., Deweindt C., Lecocq G. & Leprince D. The BTB/POZ domain: a new protein-protein interaction motif common to DNA- and actin-binding proteins. Cell Growth Differ. 6, 1193–1198 (1995). 8519696

[b19] Brown J. L. & Wu C. Repression of *Drosophila* pair-rule segmentation genes by ectopic expression of *tramtrack*. Development 117, 45–58 (1993). 8223261 10.1242/dev.117.1.45

[b20] Harrison S. D. & Travers A. A. The *tramtrack* gene encodes a *Drosophila* finger protein that interacts with the *ftz* transcriptional regulatory region and shows a novel embryonic expression pattern. EMBO J. 9, 207–216 (1990). 2104801 10.1002/j.1460-2075.1990.tb08097.x PMC551648

[b21] Read D., Levine M. & Manley J. L. Ectopic expression of the *Drosophila tramtrack* gene results in multiple embryonic defects, including repression of *even-skipped* and *fushi tarazu*. Mech. Dev. 38, 183–195 (1992). 1457380 10.1016/0925-4773(92)90052-l

[b22] Zhao C., Fu D., Dave V. & Ma J. A composite motif of the *Drosophila* morphogenetic protein bicoid critical to transcription control. J. Biol. Chem. 278, 43901–43909, doi: 10.1074/jbc.M302714200 (2003). 12939280

[b23] Lye C. M., Naylor H. W. & Sanson B. Subcellular localisations of the CPTI collection of YFP-tagged proteins in *Drosophila* embryos. Development 141, 4006–4017, doi: 10.1242/dev.111310 (2014). 25294944 PMC4197698

[b24] Childs K. S. & Goodbourn S. Identification of novel co-repressor molecules for Interferon Regulatory Factor-2. Nucleic Acids Res. 31, 3016–3026 (2003). 12799427 10.1093/nar/gkg431 PMC162335

[b25] Gamsjaeger R., Liew C. K., Loughlin F. E., Crossley M. & Mackay J. P. Sticky fingers: zinc-fingers as protein-recognition motifs. Trends in biochemical sciences 32, 63–70, doi: 10.1016/j.tibs.2006.12.007 (2007). 17210253

[b26] Wright A. P., Fox A. N., Johnson K. G. & Zinn K. Systematic screening of *Drosophila* deficiency mutations for embryonic phenotypes and orphan receptor ligands. PloS one 5, e12288, doi: 10.1371/journal.pone.0012288 (2010). 20808815 PMC2924397

[b27] Pennetta G. & Pauli D. The *Drosophila Sin3* gene encodes a widely distributed transcription factor essential for embryonic viability. Dev. Genes Evol. 208, 531–536 (1998). 9799435 10.1007/s004270050212

[b28] Zhu C. C. . *Drosophila* histone deacetylase-3 controls imaginal disc size through suppression of apoptosis. PLoS Genet. 4, e1000009, doi: 10.1371/journal.pgen.1000009 (2008). 18454196 PMC2265479

[b29] Espinas M. L. . The GAGA factor of *Drosophila* interacts with SAP18, a Sin3-associated polypeptide. EMBO Rep. 1, 253–259, doi: 10.1093/embo-reports/kvd046 (2000). 11256608 PMC1083720

[b30] Canudas S. . dSAP18 and dHDAC1 contribute to the functional regulation of the *Drosophila* Fab-7 element. Nucleic acids research 33, 4857–4864, doi: 10.1093/nar/gki776 (2005). 16135462 PMC1196206

[b31] Murawsky C. M. . Tramtrack69 interacts with the dMi-2 subunit of the *Drosophila* NuRD chromatin remodelling complex. EMBO Rep. 2, 1089–1094, doi: 10.1093/embo-reports/kve252 (2001). 11743021 PMC1084170

[b32] Swaminathan A. & Pile L. A. Regulation of cell proliferation and wing development by *Drosophila* SIN3 and String. Mech. Dev. 127, 96–106, doi: 10.1016/j.mod.2009.10.003 (2010). 19825413

[b33] Leight E. R. . Conversion of the LIN-1 ETS protein of Caenorhabditis elegans from a SUMOylated transcriptional repressor to a phosphorylated transcriptional activator. Genetics 199, 761–775, doi: 10.1534/genetics.114.172668 (2015). 25567989 PMC4349070

[b34] Reynolds N., O’Shaughnessy A. & Hendrich B. Transcriptional repressors: multifaceted regulators of gene expression. Development 140, 505–512, doi: 10.1242/dev.083105 (2013). 23293282

[b35] De Nadal E. . The MAPK Hog1 recruits Rpd3 histone deacetylase to activate osmoresponsive genes. Nature 427, 370–374, doi: 10.1038/nature02258 (2004). 14737171

[b36] Yuasa Y. . *Drosophila* homeodomain protein REPO controls glial differentiation by cooperating with ETS and BTB transcription factors. Development 130, 2419–2428 (2003). 12702656 10.1242/dev.00468

[b37] Dhordain P. . Corepressor SMRT binds the BTB/POZ repressing domain of the LAZ3/BCL6 oncoprotein. Proc. Nat. Acad. Sci. USA 94, 10762–10767 (1997). 9380707 10.1073/pnas.94.20.10762 PMC23478

[b38] Bonchuk A., Denisov S., Georgiev P. & Maksimenko O. *Drosophila* BTB/POZ domains of “ttk group” can form multimers and selectively interact with each other. J. Mol. Biol. 412, 423–436, doi: 10.1016/j.jmb.2011.07.052 (2011). 21821048

[b39] Pagans S., Pineyro D., Kosoy A., Bernues J. & Azorin F. Repression by TTK69 of GAGA-mediated activation occurs in the absence of TTK69 binding to DNA and solely requires the contribution of the POZ/BTB domain of TTK69. J. Biol. Chem. 279, 9725–9732, doi: 10.1074/jbc.M313200200 (2004). 14701830

[b40] Katada S., Imhof A. & Sassone-Corsi P. Connecting threads: epigenetics and metabolism. Cell 148, 24–28, doi: 10.1016/j.cell.2012.01.001 (2012). 22265398

[b41] Ringrose L., Rehmsmeier M., Dura J. M. & Paro R. Genome-wide prediction of Polycomb/Trithorax response elements in *Drosophila melanogaster*. Dev. Cell 5, 759–771 (2003). 14602076 10.1016/s1534-5807(03)00337-x

[b42] Xiong W. C. & Montell C. *tramtrack* is a transcriptional repressor required for cell fate determination in the *Drosophila* eye. Genes Dev. 7, 1085–1096 (1993). 8504931 10.1101/gad.7.6.1085

[b43] Greenspan R. J. Fly pushing: The theory and practice of Drosophila genetics. 2nd edn, (Cold Spring Harbor Laboratory Press, 2004).

[b44] Brown N. H. & Kafatos F. C. Functional cDNA libraries from *Drosophila* embryos. J. Mol. Biol. 203, 425–437 (1988). 3199441 10.1016/0022-2836(88)90010-1

[b45] Ito T. . Toward a protein-protein interaction map of the budding yeast: A comprehensive system to examine two-hybrid interactions in all possible combinations between the yeast proteins. Proc. Nat. Acad. Sci. USA 97, 1143–1147 (2000). 10655498 10.1073/pnas.97.3.1143 PMC15550

[b46] Ausubel F. M. . Current Protocols in Molecular Biology. (John Wiley & Sons, Inc., 1994).

[b47] Einarson M. B. & Orlinick J. R. In Protein-protein interactions (ed. Golemis E.) 37–49 (Cold Spring Harbor Laboratory Press, 2002).

[b48] Li H. H., Chiang C. S., Huang H. Y. & Liaw G. J. *mars* and *tousled-like kinase* act in parallel to ensure chromosome fidelity in *Drosophila*. J. Biomed Sci. 16, 51–63, doi: 10.1186/1423-0127-16-51 (2009). 19486529 PMC2705347

[b49] Liu N., Dansereau D. A. & Lasko P. Fat facets interacts with vasa in the *Drosophila* pole plasm and protects it from degradation. Curr. Biol. 13, 1905–1909 (2003). 14588248 10.1016/j.cub.2003.10.026

[b50] Tatsumi R. & Hattori A. Detection of giant myofibrillar proteins connectin and nebulin by electrophoresis in 2% polyacrylamide slab gels strengthened with agarose. Anal. Biochem. 224, 28–31, doi: 10.1006/abio.1995.1004 (1995). 7710083

[b51] Rothwell W. F. & Sullivan W. In Drosophila Protocols (eds Sullivan W., Ashbourner M. & Hawley R. S.) 141–157 (Cold Spring Harbor Laboratory Press, 2000).

[b52] Tautz D. & Pfeifle C. A non-radioactive *in situ* hybridization method for the localization of specific RNAs in *Drosophila* embryos reveals translational control of the segmentation gene *hunchback*. Chromosoma 98, 81–85 (1989). 2476281 10.1007/BF00291041

[b53] Chanas G., Lavrov S., Iral F., Cavalli G. & Maschat F. *Engrailed* and *polyhomeotic* maintain posterior cell identity through *cubitus-interruptus* regulation. Dev. Biol. 272, 522–535, doi: 10.1016/j.ydbio.2004.05.020 (2004). 15282166

[b54] Birch-Machin I. . Genomic analysis of heat-shock factor targets in *Drosophila*. Genome Biol. 6, R63, doi: 10.1186/gb-2005-6-7-r63 (2005). 15998452 PMC1175994

