# Supplementary material for: Pits, a protein interacting with Ttk69 and Sin3A, has links to histone deacetylation
Source: Sci Rep. 2016 Sep 13;6:33388. doi: 10.1038/srep33388 (PMC5020733; doi:10.1038/srep33388)
Supplement: Supplementary Information [file srep33388-s1.pdf]

Pits, a protein interacting with Tramtrack69 and Sin3A, has links to histone deacetylation

Gwo-Jen Liaw

Table S1. Primers used in this study

Primer name	Sequence	Cutting site
38-NF	AACGCAGCAGGTACCTCGCTGCAAAC	KpnI
38-NR	CACTGGCATAAGCTTGGGCATCTGA	HindIII
38-MR	CCACGCATCGGTACCCCACTGGACTAT	KpnI
38-MR	CCACTGGCTGCAGCGAGCCGGGTC	PstI
38-CF	CCAGTGGCGGTACCGGACAGCTGAGT	KpnI
38-CR	AAATGGACATCTAGACTCGCGCTC	XbaI
Ttk69-NF	GGTTACTAAGGGTACCTCTCAACGCTTC	KpnI
Ttk69-NR	TTCATCATGAGCTCGCGATCAAAGTC	SacI
Ttk69-CF	ATGATGAACGGTACCTCCGACCTG	KpnI
Ttk69-CR	GAGATTACTGGAGCTCAGCTGCTGGTG	SacI
PAH1-F	ACAAAAACCATGGTGAAACGCACTC	NcoI
PAH1-R	TCCGGTGAGCTCATGCACCGTTCC	SacI
PAH2-F	GTAAATCTAGGTACCCACGGCGGAGCCT	KpnI
PAH2-R	GTGATAGTCGAGCTCAAAGAGAATATCGT	SacI
PAH3-F	AGCTTTTCCGAATTCTCGAGCAAGTGC	EcoRI
PAH3-R	ACTGGGCGTAAGCTTGGCATTTCGAGC	HindIII
PAH4-F	AGGAGCCGGTGGTACCAGCTCTAGTT	KpnI
PAH4-R	TGATTGTGTGAGCTCGGCAGTATCTTCGT	SacI
tor-REF	GGGCCATAAAAACCGAAAGAA	
tor-RER	GCTGCCATTGCGAAAAATTC	
tll-F	CAACCCCTGCTGCTACTATCG	
tll-R	CCATTATTGCCGCTTTGTGAT	
actin-F	TGTGTAAAAGCACCGTGACCAT	
actin-R	CGCCATGAAGAGTGTACTTCCA	

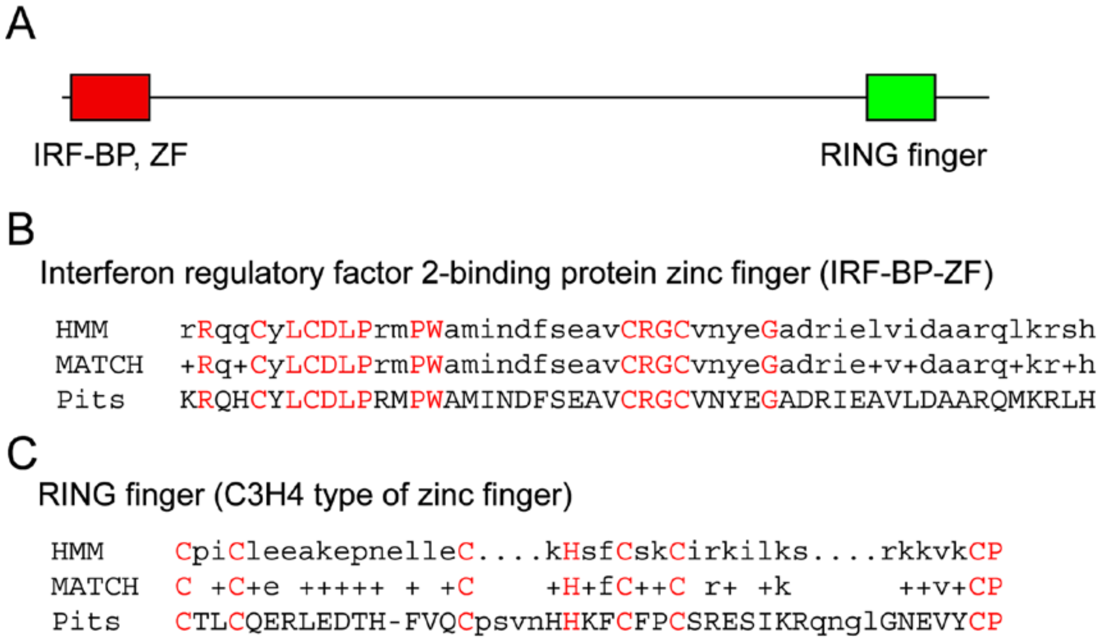

Figure S1 Two putative domains in Pits.

(A) Pfam was used to search conserved domains in Pits. The N- and C-termini of Pits contain amino acid sequences that are highly homology to the zinc-finger domain in Interferon Regulatory Factor 2-Binding Proteins (IRF-BP, red box) and Ring finger domain (C3H4 type, green box). (B, C) Alignments of amino acid sequences of Pits with the consensus sequences in Pfam (HMM). Capital letters, marked in red, indicate the highly conserved amino acid residues in the consensus sequences, which are shown in HMM logo (http://pfam.sanger.ac.uk/).

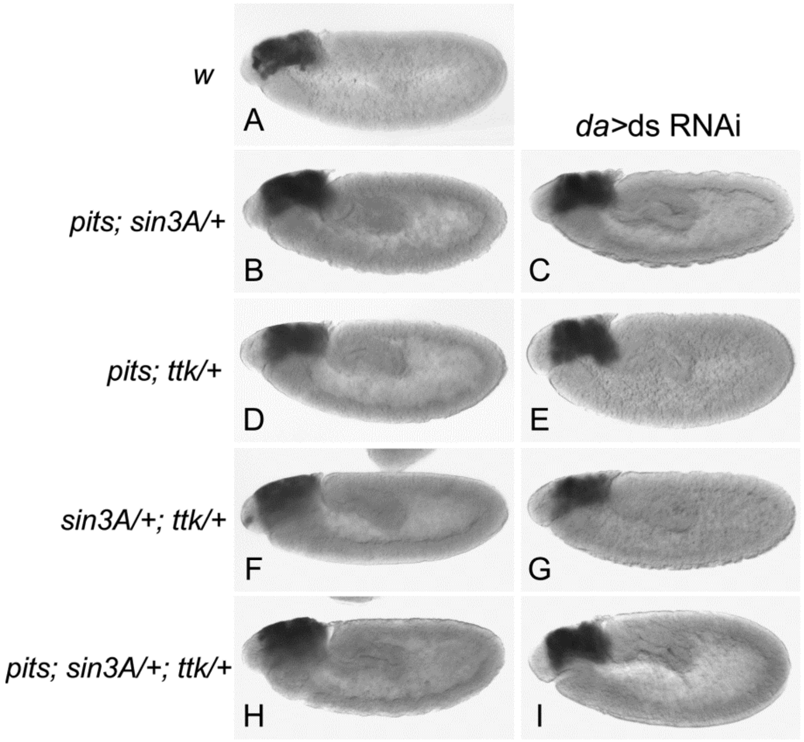

Figure S2. The expanded *tll* patterns return to normal at stage 10.

The embryos tested for the effect of the maternal and zygotic activities of *pits*, *sin3A* and/or *ttk* on *tll* expression patterns are the same as those described in Figure 5. The *tll* expression patterns were revealed by *in situ* hybridization with the digoxigenin-labeled antisense *tll* RNA. The embryos are arranged in a sagittal view, with the anterior towards the left.
